# Natural and Natural-Based Polymers: Recent Developments in Management of Emerging Pollutants

**DOI:** 10.3390/polym15092063

**Published:** 2023-04-26

**Authors:** Radu Claudiu Fierascu, Irina Fierascu, Roxana Ioana Matei (Brazdis), Doina Manaila-Maximean

**Affiliations:** 1 National Institute for Research & Development in Chemistry and Petrochemistry-ICECHIM–Bucharest, 060021 Bucharest, Romania; fierascu.radu@icechim.ro (R.C.F.); irina.fierascu@icechim.ro (I.F.);; 2 Faculty of Chemical Engineering and Biotechnologies, University “Politehnica” of Bucharest, 060042 Bucharest, Romania; 3 Faculty of Horticulture, University of Agronomic Sciences and Veterinary Medicine of Bucharest, 011464 Bucharest, Romania; 4 Faculty of Applied Sciences, University “Politehnica” of Bucharest, 060042 Bucharest, Romania; 5 Academy of Romanian Scientists, 3 Ilfov, 050044 Bucharest, Romania

**Keywords:** natural polymers, emerging pollutants, adsorption, catalytic oxidation, perspectives

## Abstract

Anthropogenic activities lead to the issue of new classes of pollutants in the environment that are not currently monitored in environmental studies. This category of pollutants (known as emerging contaminants) includes a very wide range of target substances, such as pharmaceuticals, plant protection products, personal care products, dyes, toxins, microplastics and many other industrially important intermediaries. Together with an increasing demand for clean water (both for agricultural necessities and for the increasing population consumption), the need for the removal of emerging pollutants, simultaneously with the current “green chemistry” approach, opens the door for the industrial application of natural polymers in the area of environmental protection. Recent developments in this area are presented in this paper, as well as the application of these particular natural materials for the removal of other contaminants of interest (such as radioisotopes and nanoparticles). The current knowledge regarding the processes’ kinetics is briefly presented, as well as the future development perspectives in this area.

## 1. Introduction

Nowadays, water resources are an asset which must be protected, due to the increasing imbalance between the availability and consumption of freshwater. Water pollution is a global problem affecting the lives of millions of people around the world, being a risk factor for disease and death, but also a source of life. Alongside the growth of the population and global industrialization, which contribute to high pollution in organic and inorganic compounds, climate change is another important factor that affects water resources [1]. Persistent and emerging pollutants and their degradation products are released continuously into the environment, representing a serious and global issue that has sometimes irreversible effects on human health and ecosystems. Emerging pollutants include pharmaceuticals for human or veterinary uses, products for daily care, products of industrial and household origins and pesticides, which are used in larger amounts than several decades ago, due to the increased demand for food (a consequence of the increased population).

Dealing with the difficulties of providing clean water, regardless of whether we are dealing with organic or inorganic contaminants, many current studies and technologies are being developed in order to offer reliable solutions and minimize malignant and detrimental water-borne diseases [2,3]. Classical methods, such as adsorption, precipitation, solvent extraction, co-precipitation, flocculation and coagulation, can be improved by the use of modern materials with bioactivity, biodegradability, biocompatibility and tunable physicochemical properties and morphology [4]. Activated carbon, clays, layered double hydroxide hybrids, metal or metal oxide nanoparticles (NPs) are just a few which can be nominated as good candidates for environmental applications [5,6,7,8]. Among them, biopolymers have the advantages offered by biodegradability (important from a “green chemistry” approach, although this aspect represents a subject of debate, fine-tuning of this property being necessary [9,10]) and biocompatibility, being used with success in potable and wastewater treatment [11]. The nature of the biopolymers represents one of the key parameters of an efficient treatment [12]. They are suitable for removing both types of pollutants (organic and inorganic) even if they are used as such, or in combination with other materials [13]. In addition, they offer high performance and good selectivity, and the process is cost-effective and eco-friendly [14].

In this context, the present paper aims to address the recent developments in the application of natural polymers for the management of emerging and hazardous contaminants, as well as the development perspectives in this area.

## 2. Emerging Pollutants—A New Threat to the Environment with Natural Solutions?

When addressing the issue of emerging pollutants, one would usually think of newly synthesized chemicals or compounds. However, the term relates more to the difficulties encountered in their monitoring, due to technological drawbacks or legal oversights, and less to the nature of these pollutants. As a generally accepted definition, emerging pollutants (EPs) are considered chemicals (natural or synthetic) **not commonly monitored** in the environment, having the potential to cause detrimental effects on ecosystems or human health [15]. As can be seen, the classification of a particular substance as an emerging pollutant is more related to the lack of routine monitoring and less to their nature. Although many of these pollutants are indeed newly synthesized chemicals, there are different types of EPs naturally occurring and already present in the environment, their potential effects being only recently evaluated.

According to the NORMAN network [16], EPs can be classified into 21 groups, covering very different types of substances, from biocides and plant protection chemicals to drugs, pharmaceutical and personal care products, and from smoke compounds to surfactants and human metabolites. Their origins are also very different depending on their nature. Most of these pollutants originate from industrial processes or human activities (not being addressed by urban or industrial treatment plants), but can also be traced to diffuse sources, such as atmospheric deposition or crop/animal production [15]. For the present work, the NORMAN emerging pollutants database was used as a reference [16].

The area of EPs has represented a domain of interest for over a decade now, with a series of very interesting opinions, review works and books being published on this topic, which we recommend for future reading [17,18,19,20,21,22,23].

One of the interesting approaches regarding the removal of emerging pollutants is represented by the use of natural polymers. In a world where the continuous diminishing of resources and environmental pollution have become critical issues, the use of easily recyclable or available materials becomes vital.

To have clear insight into polymers, their constituents and properties, polymers have been classified according to several points of view [24]. One of these criteria is represented by their origins [25]. According to this classification, *natural polymers* (formed by proteins, nucleic acids, polysaccharides or polyhydrocarbons), *artificial polymers* (obtained by modifying natural ones; for example, viscose and cellophane) and *synthetic polymers* can be found, obtained by chemical reactions starting from monomers.

The main EU law to protect human health and the environment from the risks that can be posed by chemicals is the **“Registration, evaluation, authorization and restriction of chemicals”** (REACH) [26]. In accordance with REACH (Article 3(5)), a polymer is defined as a substance meeting the following criteria: more than 50% of the weight of that substance consists of polymer molecules, and the amount of polymer molecules presenting the same molecular weight must be less than 50% of the weight of the substance. According to ECHA, natural polymers are polymers that are the result of a polymerization process that has taken place in nature, independently of the extraction process. It results that natural polymers are not necessarily “substances which occur in nature”. Considering these differences, some authors are introducing in their research the term of “naturally occurring polymers” [27].

In [25], Doppalapudi, considering the origin of natural polymers, systematizes them into three main classes: natural polymers of plant origin, of animal origin and of microbial origin, with polysaccharides being present in each class mentioned.

Polysaccharides of plant origin are cellulose, hemicellulose, cyclodextrins, starch, inulin, pectin, glucomannan, guar gum, arabinogalactan and carrageenan. Animal-origin polysaccharides include chitosan, hyaluronan and chondroitin sulfate. Microbial-origin polysaccharides comprise alginate and dextran.

Proteins are mainly of animal origin, such as collagen, gelatin, albumin, fibrin and silk fibroin; there are also proteins of vegetable origin, such as soy. Of plant origin are shellac resin and some esters; other esters (polyhydroxyalkanoate) have a microbial genesis, together with polyamides (polyglutamate) and polyanhydrides (polyphosphate).

The naturally occurring polymers have also been classified into six main categories [27,28]: polysaccharides (starch, cellulose, chitin, pectin, alginic acid, natural gums, etc.), one of the most studied groups of natural polymers [29,30,31]; proteins or naturally occurring polyamides found in animal and vegetable sources; polyisoprenes or natural rubbers and similar materials isolated from saps of plants; polynucleotides, which comprise DNA and RNA found in all living organisms; lignin or polymeric materials of coniferyl alcohol and related substances; naturally occurring miscellaneous polymers, such as shellac, a resin secreted by the lac insect.

***Polysaccharides*** are of various types depending on their structure or function [32,33]. In terms of function, there are three main types: storage polysaccharides such as starch and glycogen, structural polysaccharides such as cellulose and chitin, and gel-forming polysaccharides such as alginic acid and mucopolysaccharides [32].

The following sections present the possible applications of natural polymers for the removal of emerging pollutants, the information being grouped considering the targeted pollutant. In addition, we chose to include in the current review examples of the use of chitosan. Although not technically a natural polymer (according to the definitions provided above), chitosan, due to its easy production (simple deacetylation of natural chitin), non-toxic characteristics, as well as its wide application in different important areas, chitosan is often included in the class of “natural polymers” [34,35,36].

## 3. Methodology

For the selection of the studies to be included in the present review, we followed the recommendations of Preferred Reporting Items for Systematic Reviews and Meta-Analyses 2020 (PRISMA) [37]. The research strategy was formulated according to the PICO (Problem, Intervention, Comparison, Outcome) approach (Table 1).

The research was conducted based on the PICO question: “Can natural polymers be successfully used in the management of emerging pollutants?” As such, the following inclusion/exclusion criteria were defined.

Inclusion criteria:

- Research articles published from 2012 to the present, full text;
- Articles published or available in English;
- Removal of emerging pollutants—for the automatic screening, only the term “pollutant” was used;
- Evaluation of natural polymers’ application in the management of emerging pollutants (as defined in Section 2)—manual screening;
- Relevance to the review topic (new information provided).

Exclusion criteria:

- Articles published before 2012;
- Book chapters or book;
- Review or systematic review articles;
- Conference papers, notes, letters, short surveys, errata or conference reviews;
- Articles published in languages other than English;
- Articles presenting the removal of pollutants monitored in routine studies (such as commonly encountered heavy metals).

The literature search was conducted using the database SCOPUS (as an exhaustive literature database), using “natural polymer” as the primary search term. A further selection of the articles was performed automatically, using the inclusion/exclusion criteria defined above, and inclusion in the present review was decided after a full reading of the manuscript.

## 4. Results

**Figure 1:**
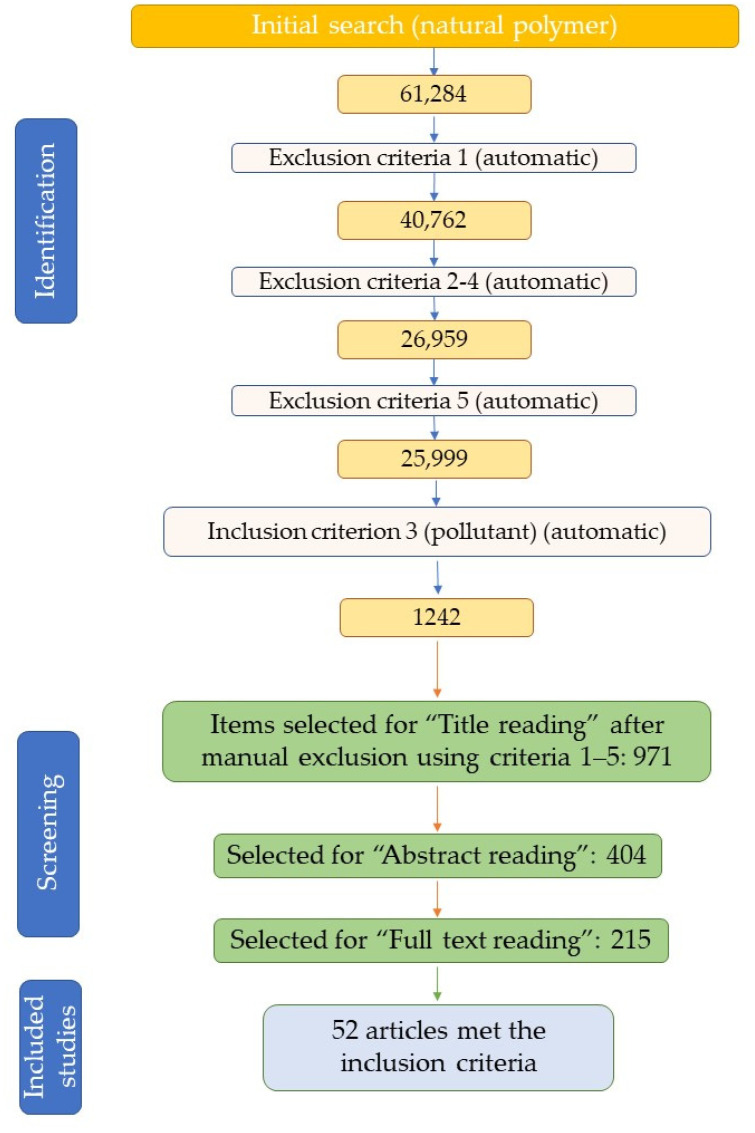
Article selection process flowchart.

After applying the entire set of exclusion/inclusion criteria, followed by title, abstract and full-text reading, a total of 52 articles (and one correction article) were selected for inclusion in the present review (Figure 1), covering the removal of emerging pollutants using natural polymers. To the selected articles, other works were added for providing the necessary context. These articles were retrieved by a “search and find”/manual selection approach using the SCOPUS database (by searching using specific keywords), or were suggested by the reviewers during the peer review process. For the purpose of the current review, studies presenting the removal of heavy metals (already routinely monitored) and water remediation studies (i.e., turbidity remediation, sterilization, oil adsorption, etc.) were not considered for inclusion.

**Figure 2:**
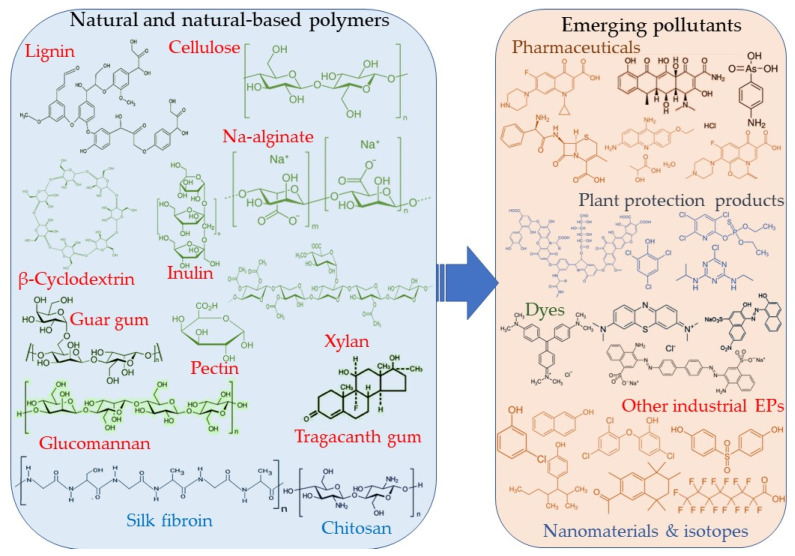
Natural and natural-based polymers and main contaminants discussed in the present review work.

The works selected for screening present the application of natural (and one natural-based) polymers for the management of emerging pollutants and some other emerging substances (potentially hazardous). Figure 2 presents the natural polymers reviewed and the major pollutant categories considered for the literature study.

## 5. Natural Polymers for the Management of Emerging Pollutants

### 5.1. Management of Pharmaceuticals Using Natural Polymers

One of the most diverse groups of EPs is represented by pharmaceutical substances. Most often originating from patient excretion, the presence of pharmaceutical substances as an environmental pollutant can also be related to emissions during production processes, as well as from the incorrect disposal of unneeded substances [38]. As the pharmaceutical industry represents an ever-growing industry, it can be expected that pharmaceutical pollution will follow a similar trend. At the same time, the pandemic conditions in the past few years led to a spike in the use of over-the-counter and prescription drugs, causing an increase in their presence in the environment, as well as the appearance of some new substances [39]. The pharmaceuticals present in the environment can cause a wide range of health issues; i.e., some of them act as endocrine disruptors (some being used as intentional endocrine disruptors), while others can contribute to the development of antimicrobial resistance [40]. Due to the varied nature of pharmaceutical compounds, there is a correspondingly high number of studies regarding their management. Among them, the literature survey identified several using natural polymers, presented in Table 2.

The examples above provide a good glimpse of the potential applications of natural polymers for the removal of pharmaceuticals. From the examples presented, a natural trend to use easily available resources for the removal of hazardous pollutants can be observed. For instance, two of the most common and often used natural polymers (in different applications), chitosan and sodium alginate, were evaluated by Sahin et al. [46] for the development of composite beads with magnetic nanoparticles (also synthesized by green routes—phytosynthesis using *Lathyrus brachypterus* extract). The resulting composites, providing a mixture of beneficial properties of magnetic nanoparticles (high catalyst loading capacities, dispersion properties, stability, recyclability, surface area and separability) and natural polymers’ up-take capacity, showed high efficiency for the removal of two nonsteroidal anti-inflammatory drugs (naproxen and diclofenac), both in individual and simultaneous experiments. The best results were obtained by the authors using the bimetallic nanoparticles deposited on chitosan (for diclofenac removal) and alginate (for naproxen removal). The results obtained were comparable with literature data but obtained using much shorter reaction times (9 min compared with over 60 min in other studies). Additionally, the conclusion of the authors was that the process was more inclined towards physisorption (kinetics respecting the pseudo-first-order model). A physical adsorption (explained by Van der Waals forces) was described by the authors as the primary adsorption mechanism. Rusu et al. evaluated the removal of acrinol (also known by the trade name Rivanol) using composites obtained from residual microbial biomass (*Saccharomyces pastorianus*) and a natural polymer (sodium alginate, modified by the addition of calcium chloride), both in batch [47] and in fixed-bed column studies [48]. The authors proved that the developed composites can be successfully applied for theoretical studies (using batch experiments) and can also be practically applied in scaled-up installations, as the fixed-bed column experiments would suggest.

Some of the cited authors also provide some insights into the mechanisms involved in the contaminants’ management, as well as in the interaction between natural polymers and contaminants. For example, Balachandramohan and Sivasankar [41] attribute the efficiency of ciprofloxacin adsorption to the electrostatic interaction between the negative surface of the natural polymer composite and the positively charged compound, while the adsorption of doxycycline hydrochloride on a lignin xanthate resin–bentonite clay composite was attributed by Kong et al. [42] to the interactions between the benzene ring in the contaminant and lignin moieties, as well as to hydrogen bonds appearing between the two compounds, suggesting the apparition of a macromolecular complex, as also observed by Peng et al. [43] for the adsorption of p-arsanilic acid on ionic liquid-modified cellulose, and by Rusu et al. [44] for the adsorption of cephalexin on *Saccharomyces cerevisiae* calcium alginate beads.

Generally, pharmaceutical removal using natural polymer processes is governed by the pseudo-second-order model (the process being more inclined towards physisorption), while the best-fitting models for the equilibrium isotherms were the Freundlich and Langmuir models, supporting the authors’ claims towards multi-layer adsorption on heterogenous surfaces and monolayer adsorption, respectively. Another relevant aspect that arises from literature data is that natural polymers are not commonly used as single adsorbents, but are more likely to be used in composites, with either other organic or inorganic compounds (which could act either as adsorbents or catalysts), as is the case for all the examples presented in Table 2.

### 5.2. Management of Plant Protection Product Pollutants Using Natural Polymers

With the increase in the global human population and the rising food demand, the use of pesticides and fungicides has seen an unprecedented spread. Although very useful in providing food in the quantities and at the quality required, pesticides and fungicides can have different adverse effects on water quality, and, consequently, on ecosystems and human health [49]. Although the effects of pesticides and fungicides are under continuous scrutiny, their use is majorly based on industry-performed studies, as other authors noticed [50]. Furthermore, their long-term effects on ecosystems and human health still represent a subject of debate. As such, there is a continuous need to develop appropriate technologies and materials for the removal of pesticides and fungicides from the environment. Some of the pesticides and fungicides cannot be defined as “emerging pollutants”, strictly speaking, as there are national and/or international regulations requiring their monitoring. However, some examples are presented that can represent the basis for the development of new technologies for other compounds that are not currently regulated (i.e., phosphate).

Recently, the use of so-called “biostimulants” raised the interest of both the scientific community and farmers, leading to an increase in their practical use. Biostimulants can be defined as “a product stimulating plant nutrition processes independently of the product’s nutrient content with the sole aim of improving one or more of the following characteristics of the plant or the plant rhizosphere: nutrient use efficiency; tolerance to abiotic stress; quality traits; availability of confined nutrients in soil or rhizosphere” [51,52]. Obviously, such a comprehensive definition leads to a very wide range of substances and compounds, their classification in this group being based on their final effect on plants and not their nature and/or composition. The class is currently under scrutiny for regulation at the European level, with an EU regulation recently being adopted covering this aspect [52].

Table 3 summarizes some examples of natural polymers applied for the removal of pesticides, fungicides and biostimulants from the environment.

One interesting approach on this topic is presented by Attallah et al. [58]. Using natural and natural-based polymers (gelatin and chitosan), the authors developed a polymer composite with a removal efficiency of 87.13% to 94.48% for atrazine and 82.65% to 96.45% for fenitrothion after 120 min, superior to organic and inorganic adsorbents, as presented by literature data. The authors also proposed adsorption mechanisms, in which atrazine is removed by adsorption onto the composite pores and to the hydrophilic sites of oxygen-containing groups by hydrogen bonding, while fenitrothion is adsorbed on the surface of the composite and to the hydrophobic sites’ interactions through π–π stacking. The mechanisms would allow the use of the developed composites in real conditions (in the presence of multiple target pollutants).

Humic acid’s adsorption was attributed to the electrostatic interactions between the contaminant and the natural polymer part of the composites [53,54] and by ion exchange [57], a mechanism also involved in the adsorption of fluvic acid. Ion exchange was also described as the main mechanism in the adsorption of phosphate ions [55,56].

### 5.3. Management of Industrial Dyes and Dye Models Using Natural Polymers

The development of the textile industry has led to the extensive use of synthetic dyes, which could reach water sources as industrial effluents. Generally speaking, dyes are non-biodegradable organic compounds that have coloring properties towards a given substrate (due to the presence of chromophoric groups in their structure) [61]. As in many developing countries, the effluents (often insufficiently treated) are present in agricultural irrigation water [61], the dyes often reach water sources [62,63], having toxic, mutagenic and carcinogenic effects, while also impairing the photosynthesis process by preventing light penetration through water. The dyes have the tendency to bioaccumulate in superior organisms by crossing the food chain [61]. Of specific interest are azo-dyes, which have a relatively low binding yield during the industrial process (with up to 50% being released as wastewater). Other types of dyes can emerge from different industrial applications, including histological applications, biotechnology or the food and beverage industry). In practice, studies often use model dyes, i.e., methylene blue, a cationic and primary thiazine dye. Although it has high toxicity and a wide range of applications [64], methylene blue (MB) is easily photocatalyzed. As such, in the present work, MB is presented as a model for the removal of similar dyes, and not as a pollutant.

Considering their extensive use and potentially harmful effects, the removal of dyes from water streams represents a current subject of interest. An overview of the current applications of natural polymers for the removal of dyes and dye models is presented in Table 4.

One of the most encountered natural polymers (chitosan) was also demonstrated by Alshahrani et al. [84] to form heterostructures with hydroxyapatite and cerium oxide, which proved efficient (11–96% removal efficiencies) in the removal of different industrially important dyes (Brilliant blue, Congo red, crystal violet, methylene blue, methyl orange, Rhodamine B).

Unlike other types of pollutants, the dyes can be removed by different methods, including filtration, adsorption and (photo)degradation (as presented in Table 4). Among the presented examples, the most encountered natural polymers used are polysaccharides, not only of vegetal origin (including cellulose, xylan, lignin, guar gum, β-cyclodextrin, pectin, glucomannan, Tragacanth gum, sodium alginate) but also of animal origin (chitosan) and proteins (silk).

The materials developed for dye removal include foams and sponges, hydrogels and microgels, membranes, as well as polymeric beads and (nano)composites. As a general remark, the adsorption model for their use is found by most authors to follow the Langmuir model, but the Freundlich model is also presented (for hydrogels and composites containing alginate or silk). Additionally, Huang et al. [71] suggested the Sips model for the adsorption of MB on citric acid-crosslinked β-cyclodextrin. Regarding the kinetics of the processes, the examples presented equally support pseudo-first-order and pseudo-second-order kinetics. However, it can be observed that for protein and natural polymers of animal origin (chitosan), the predominant model is represented by the pseudo-first-order model (reaction rate depending only on one reactant concentration), while for the natural polymers of vegetal origin, the predominant kinetic model proposed by the authors is the pseudo-second-order model (a model which proposes chemisorption as the rate-limiting step [85]). The exceptions to this model are represented by the adsorption of malachite green and MB on Konjac glucomannan/graphene oxide sponges and on hydrogel tragacanth gum/CaCO_3_ nanoparticles, respectively [75], and the degradation of MB using Ca-alginate/CuO beads [81]. Several of the presented studies (see Table 4) demonstrate the positive influence of the natural polymer used on the removal efficiency of the final composite, as well as a superior removal capacity, compared with traditional materials.

The removal of the dyes by adsorption is usually achieved by electrostatic interactions (as demonstrated in several studies [66,71,72,73,78,79,80,82]), as well as the adsorption on both the surface and in the mesopores/macropores of composites [75,76]. On the other hand, the photodegradation of dyes using the proposed composites involves the use of active materials (such as TiO_2_, Fe^0^, ZnO), which leads to the formation of active species (reactive oxygen species •OH, electrons) involved in the photodegradation mechanisms [41,65,76], while flocculation is achieved by the charge neutralization mechanism [70]. The general mechanisms (and the interaction between the contaminants and the composites) are strongly dependent on the nature of the dyes used in the studies, as well as on the contaminant removal method selected (adsorption, photodegradation, flocculation).

### 5.4. Management of Other Types of Industrial Emerging Pollutants Using Natural Polymers

Other types of emerging pollutants currently under scrutiny are represented by personal care products, plasticizers, surfactants and other types of industrially important chemical materials. Some examples of their removal are presented in Table 5.

A particular case is represented by the very interesting study of Ling et al. [93]. In their study, the authors evaluated the capacity of porous β-cyclodextrin polymers for the removal of 90 micropollutants (ranging from pesticides and industrial compounds to pharmaceuticals and personal care products, at 10 mg/L concentration), compared with a known adsorbent, coconut shell-activated carbon, in batch and flow-through experiments. The main conclusion of the batch experiments was that compared with the activated carbon process, the adsorption using the natural polymer was faster, but selective (for some of the pollutants, complete uptake was realized in 5 min). The authors correlated the selectivity of the natural polymer with the McGowan volume (bulk size of 1 mol of molecules) and the charge state of the target compounds. Overall, the natural polymer seemed to be a very good alternative to activated carbon, especially for pollutants with higher McGowan volume and positive charge (although for neutral and negatively charged pollutants with McGowan volumes > 1.7 (cm^3^/mol) × 100, the adsorbent also had very good results).

Similar results were obtained by Topuz et al. [94] in the removal of several dyes and polycyclic aromatic hydrocarbons using nanofibrous hyper-crosslinked cyclodextrin network membranes produced by electrospinning, 460 nm in diameter. The results obtained for most of the micropollutants are superior to literature data, and the membranes exhibited very good reusability after acidic methanol treatment.

A widely encountered class of chemicals (personal care products, defined as products used to increase the quality of life), often associated with pharmaceuticals in the wider class of pharmaceuticals and personal care products (PPCP), is currently being defined as one of the most harmful chemicals to ecosystems, being encountered all over the world [95]. Examples of such contaminants related to PCPs include triclosan (antimicrobial agent) and tonalide (fragrance), commonly used in cosmetics. Merlo et al. [59] presented their extraction using polymeric films developed by incorporating different plasticizers in cellulose triacetate by solvent casting, with 93–94% efficiency after 8 h, a higher efficiency than the one recorded for the insecticide chlorpyrifos (see Table 2).

Another industry from which a significant class of emerging pollutants (surfactants) are discharged is the cleaning products industry. An example of a surfactant that was successfully eliminated using natural polymers is perfluorooctanoic acid, adsorbed using β-cyclodextrin-based polymers [88].

The plastics industry is also one of the industries to which hazardous emerging pollutants can be traced. Plasticizers (such as phthalic anhydride), intermediaries or additives (such as bisphenol A) or curing agents in the epoxy industry (such as bisphenol S) were successfully removed using chitosan or β-cyclodextrin composites. Other examples of industrial emerging pollutant management presented in Table 5 include the industrial intermediates chlorophenol (adsorbed using chitosan-based composites), nonylphenol (removed by flocculation using carboxymethylcellulose composites), 2-naphthol, 3-phenylphenol (adsorbed using β-cyclodextrin polymeric composites), 2-nitroaniline, p-nitrophenol (removed by catalytic reduction using chitosan-based hydrogels or composites, as well as calcium alginate/inorganic beads), phenol, p-cresol, catechol and 2-aminophenol (biodegradation using a mixture of chitosan-based composites and microorganisms). In degradation studies, the kinetics followed a pseudo-first-order model, and the results were superior to the literature data. Kinetic studies were also performed for the adsorption of different emerging pollutants on β-cyclodextrin-based polymers, with the two evaluated studies revealing a pseudo-second-order model. Composites based on the same natural polymer exhibited adsorption isotherms fitting the Freundlich model (for the adsorption of perfluorooctanoic acid, bisphenol A, 2-naphthol, 3-phenylphenol and bisphenol S) and the Langmuir model for the adsorption of bisphenol A on citric acid-crosslinked β-cyclodextrin [71].

Regarding the mechanisms involved in the contaminants’ management, Hu et al. [57] attributed the removal of bisphenol A on β-cyclodextrin composites to the adsorption in the polymer’s cavities and other hydrophobic sites by hydrophobic interactions; a similar mechanism was proposed by Huang et al. [71] and Sun et al. [60]. For the flocculation removal of nonylphenol, Yang et al. [87] proposed a mechanism in which charge attraction and hydrophobic interaction play key roles. As previously presented for industrial dyes, the degradation of the contaminants presented in Table 5 was achieved via electron transfer [89,91]. The general mechanisms proposed for the degradation of phenolic compounds involve the adsorption of contaminants on the surface of the composite, electron transfer and adsorption of degradation products on the surface of the composite [81,92].

### 5.5. Management of Other Hazardous Emerging Pollutants Using Natural Polymers

Besides the examples discussed in the previous sections (not intended as an exhaustive presentation), there are numerous other types of chemicals belonging to the class of emerging contaminants. For example, polycyclic aromatic hydrocarbons (PAHs) are encountered across the world, caused especially by long-term anthropogenic sources. PAHs are continuously considered a major concern, as their properties (structural, hydrophobicity, thermostability, among others) are correlated with toxic, mutagenic or carcinogenic effects [92]. Some very interesting review works were recently published on this topic, which we suggest for further reading [96,97,98].

In the same category of hazardous EPs, microplastics, toxins or radionuclides can also be included. Table 6 presents some examples of the use of natural polymers for the management of such pollutants.

The use of cellulose-based compounds, as presented in Table 6, is a viable approach for the removal of PAHs from water sources (as demonstrated by Mehmandost et al. [100], who suggested a removal mechanism based on PAHs/polymer π–π interactions), as well as from mainstream cigarette smoke. The second application would be of particular importance for avoiding smoke-related illnesses [101].

A potential application of a granulated resin obtained using natrium alginate as a natural polymer precursor is represented by the chelation of radioisotope models, such as U(VI) ions, as presented by Hassan [104]. As in the case of the composite sponges made of Konjac glucomannan/graphene oxide, presented by Chen et al. [75], the polysaccharide composites exhibited high affinity for the isotope model, with a similar adsorption capacity. The proposed mechanism was chelation using the attraction forces between the opposite charges of the natural compound and the cationic metal.

With the unprecedented development of nanotechnology, nanoparticles will soon become emerging pollutants of high interest. Although the potentially detrimental effects of nanoparticles are, in most cases, a subject of debate, their particularities (small dimensions, non-biodegradability, mobility, high surface areas, etc.), differentiate them from the corresponding ions (which are in some cases currently monitored), making them a preventive subject for environmental management studies. Natural polymers can be used in this area, as demonstrated for the rejection of gold nanoparticles using silk membranes or the removal of TiO_2_ nanoparticles by flocculation using polysaccharide composites.

More recently included in the category of emerging pollutants [106,107], microplastics and toxins can also be successfully managed by the use of natural polymers. Present in a large variety of products or resulting from the breakdown of larger materials, microplastics became a global issue due to their easy crossing through the food chain, ultimately affecting human health (although currently, their effects are poorly understood) [108]. Asma and Valiyaveettil [102] demonstrated the adsorption by electrostatic interactions of different types of micro- and nanoplastics using cellulose surface functionalized with polyethylenimine with more than 97% efficiency, the adsorption kinetics following a pseudo-second-order model.

Naturally occurring (being produced by cyanobacteria) hepatotoxic cyanotoxin microcystins (MCs) are currently considered emerging contaminants, with a poorly understood ecological role. The cyanotoxins were proven to have toxic effects on ecosystems [109] and human health [110]. In this context, Gomez-Maldonado et al. [103] evaluated the adsorption of microcystin-LR (the most toxic microcystin) at different concentrations (1.5 and 0.8 µg/mL) using cellulose–β-cyclodextrin aerogels. The aerogels revealed a good adsorption capacity (0.078 mg/g) and the kinetics studied revealed that the process fit a pseudo-second-order model, allowing the proposal of a viable alternative for the improvement of water quality. According to the authors, the adsorption process targets the hydrophobic Adda groups in the microcystin.

Another very useful potential application of natural polymers is the detection of hazardous phenolic compounds, as demonstrated by Balram et al. [91]. Besides the efficient degradation of p-nitrophenol, the nanocomposite, comprising chitosan-wrapped carbon nanofibers embedded with Ag-doped Co_3_O_4_ deposited onto a carbon electrode, also proved efficient in the detection of the same phenolic compound by electrochemical methods. The sensor developed with the composite revealed a sensitivity of 55.98 μAμM^−1^cm^−2^ and a detection limit of 0.4 nM; its application was demonstrated using sewage, underground water and tomatoes.

## 6. Concluding Remarks and Future Perspectives

The use of natural polymers for the management of emerging pollutants represents a topic of interest worldwide. Their availability in renewable resources, ease of manufacturing composites, as well as the possibilities to develop truly “green” materials with minimum environmental impact make them very good candidates for developing alternative de-pollution technologies. At the same time, their recyclability and stability also represent tremendous advantages.

According to the literature data, there are some steps necessary before the industrial development of these technologies. Unlike other newly proposed technologies, the reaction mechanisms are rather well established. For example, the adsorption mechanisms of β-cyclodextrin (one of the most encountered materials among the natural polymers applied in environmental studies) are presented in many studies. For model dyes (such as methylene blue), the adsorption most probably occurs primarily by surface binding, while for a non-polar molecule (such as bisphenol A), the removal is achieved by host–guest inclusion in the hydrophobic cavities [71]. Another non-competitive adsorption process was observed by Hu et al. [57] for the removal of humic acid and bisphenol A using a β-cyclodextrin polymer (humic acid being removed by ion exchange with the quaternary ammonium groups of the polymer, while bisphenol A adsorbed in the cyclodextrin cavities by hydrophobic interactions). The different mechanisms would allow the independent removal of both types of pollutants from complex matrices. The use of this particular heptasaccharide in de-pollution studies proved to be efficient at a laboratory scale, and has several advantages such as the ease of preparation (performed by the action of cycloglycosyltransferase enzyme on partially hydrolyzed starch [111]) and low production costs. However, the upscaling to industrial applications is complicated by relatively complex functionalization steps, which are often required in order to reach appropriate decontamination properties [112]. A possible response to this issue could be represented by the use of citric acid-crosslinked β-cyclodextrin, demonstrated to have adsorptive properties towards dyes and phenolic compounds [71]. More widespread materials (including sodium alginate in its modified calcium form, cellulose or chitosan) could be viable alternatives, as they possess adsorptive properties, even in unmodified form or with basic alteration of their native form [43,80,81]. Such materials (iron oxide nanoparticle-loaded chitosan composites) proved successful, e.g., in the pilot-scale adsorption of phosphate [51].

On the other hand, when functionalized with different types of other compounds, all natural polymers proved effective in the removal of pollutants, either by adsorption, catalytic oxidation or flocculation. The efficiency of the process usually depends on other constituents of the composites, although the polymers were proven to contribute to the final effect (as demonstrated for lignin and chitosan in the adsorption of methylene blue, and Acid Blue-113, respectively [72,78], by comparing the results obtained with and without the natural polymer). However, in many of the presented studies, the natural polymer is used as a support material. This raises some questions regarding whose responses actually could constitute the future of this particular removal method: can the natural polymers be used, with as little as possible modification from their native state for the industrial-scale removal of emerging contaminants? This remains a question to be answered in future studies, as the field of emerging contaminants will unfortunately provide continuous research topics.

In our opinion, future studies should focus on the aspect of practical, industrial application, considering all involved factors: associated costs to produce the active material, energetic costs or costs related to the regeneration of the active materials, alongside the efficiency of the developed technologies, compared to the current state of the art (i.e., the use of activated carbon, clays, etc.) [113,114].

## Figures and Tables

**Table 1:** Definition of PICO strategy applied in the present work.

P (Problem)	Presence of emerging pollutants in water streams
I (Intervention)	Application of innovative, natural polymeric materials for emerging pollutants’ removal
C (Comparison)	Materials with known properties in environment protection or singular components, in the case of composite materials
O (Outcome)	Improvement of pollutant uptake and the use of natural polymers

**Table 2:** Management of pharmaceutical EPs using natural polymers (in chronological order).

Natural Polymer	Targeted Application	Application Form	Obtained Results	Process Parameters	Ref.
Guar gum	Ciprofloxacin (fluoroquinolone antibiotic) adsorption, 15 mg/L	Zerovalent iron-guar gum nanocomposite, spherical, particle size of ∼60–70 nm (0.5 g/L)	94% removal after 60 min	Superior adsorption at pH 4, compared with pH 2.6 (41%), superior to zerovalent ion by itself (69%); removal efficiency over 85% after 2 recycling cycles	[41]
Lignin	Adsorption of doxycycline hydrochloride (tetracycline antibiotic), 30 mg/L	Lignin xanthate resin–bentonite clay composite, porous structure	Adsorption capacity 438.75 mg/g	Superior to bentonite (119.93 mg/g); isotherm—Langmuir model (monolayer and uniform adsorption); kinetics—pseudo-second-order model (multilayer adsorption on heterogeneous surfaces, adsorption through electrostatic interaction, hydrogen bonding and π–π interactions—chemisorption)	[42]
Cellulose	p-arsanilic acid (veterinary use)	Ionic liquid-modified cellulose	Adsorption capacity 216.9 mg/g	Comparable results with other works, fast adsorption rate 3.21 × 10^−3^ g/(mg × min), process conformed to Freundlich (multi-layer adsorption on heterogenous surfaces) and pseudo-second-order kinetics model; 77% of initial capacity after six recycling cycles	[43]
Sodium alginate	Cephalexin (beta-lactam antibiotic), 30 mg/L	*Saccharomyces cerevisiae*/calcium alginate composite beads, 3.32 mm diameter	Adsorption capacity 94.34 mg/g	Superior to literature biosorbents; isotherm—Langmuir model; kinetics—pseudo-second-order model	[44]
Inulin	Adsorption and heterogenous catalysis of ofloxacin (fluoroquinolone-type antibiotic), 25 mM	Immobilization of laccase onto dialdehyde inulin -coated silica-capped magnetite nanoparticles, 90% of the particles with diameters 1–10 nm	63% removal capacity	Superior results to laccase alone. Ofloxacin removal—through adsorption and biodegradation mechanisms; kinetics—pseudo-second-order model; 50% of initial activity after 6 reuse cycles	[45]
Chitosan, sodium alginate	Adsorption and catalytic oxidation of naproxen, diclofenac (nonsteroidal anti-inflammatory drugs—NSAID), 25 mg/L, individually or simultaneously	Magnetite or Fe/Cu nanoparticles, on alginate or chitosan beads	84/92% removal of naproxen/diclofenac, using Fe/Cu on alginate or chitosan, respectively, after 9 min	Good removal capacity in short reaction time (9 min); kinetics—pseudo-first-order model (reaction more inclined towards physisorption); slight decrease in removal efficiencies after three recycling cycles	[46]
Sodium alginate	Biosorption of ethacridine lactate (aromatic organic compound, antiseptic, trade name Rivanol), 50 mg/L	Encapsulation of *Saccharomyces pastorianus* residual biomass in calcium alginate, irregular and elongated shape, 1.89 mm diameter	Maximum capacity 21.39 mg/g, batch study	Kinetics—pseudo-second-order and intraparticle diffusion (adsorption controlled by one of the following: film diffusion/adsorbate diffusion into the pores/ surface adsorption); equilibrium isotherms—Freundlich and Dubinin–Radushkevich (adsorption is related to pores volume filling).	[47]
Sodium alginate	Biosorption of ethacridine lactate (aromatic organic compound, antiseptic, trade name Rivanol), 20/40 mg/L	Encapsulation of *Saccharomyces pastorianus* residual biomass in calcium alginate; spherical, whitish beads, 3.218 mm diameter	Biosorption capacity 138.584 mg/g, fixed-bed column study	Biosorption capacity variable with bed height, pollutant concentration and flow rate. Experimental data best fit the Yoon–Nelson (premise—decreasing adsorption rate directly proportional to adsorbate adsorption), Clark (premise—adsorption is mass-transfer concept combined with Freundlich isotherm, piston type behavior of flow in column), and, to a lesser extent, Yan model (dose–response model)	[48]

**Table 3:** Management of plant protection products using natural polymers (in chronological order).

Natural Polymer	Pollutant	Pollutant Class	Application Form	Obtained Results	Process Parameters	Ref.
Cellulose	Humic acid, 1 g/L	Biostimulant	Cellulose nanofibers obtained by addition of epoxypropyl trimethyl ammonium chloride at 65 °C, 8 h	Adsorption rate 184 × 10^−3^ min^−1^	Composite (1 mL, 0.2–0.4 wt%) added to different amounts of humic acid solution, shaken for 2 days; best results at pH 4.5; at pH = 6.2, adsorption rate = 49 × 10^−3^ min^−1^; isotherms—Langmuir model	[53]
Chitosan	Humic acid	Biostimulant	Polyacrylamide/chitosan semi-interpenetrating network hydrogels	Maximum adsorption 166.30 mg/g	0.025 g of composite dispersed in 50 mL of 60 mg/mL pollutant; pH = 3 to 11; results superior to other organic and inorganic adsorbents in literature; isotherm—Sips model (multilayer adsorption on heterogeneous surfaces at low concentrations and monolayer adsorption at higher pollutant concentrations)	[54]
Chitosan	Phosphate	Fungicide	Iron oxide nanoparticle-loaded chitosan composites	Removal capacity of 0.059 mgP/g	Pilot plant: adsorption tower (height = 100 cm, inner diameter = 45 cm, flow rate = 7.05 ± 0.18 L/min), composite volume = 80 L, composite weight = 85.74 kg; constant adsorption capacity after six recycling cycles	[55]
Sodium alginate	Phosphate	Fungicide	Iron crosslinked alginate beads	Maximum adsorption capacity 79 mg/g	Experiments conducted with synthetic water; kinetics—pseudo-second-order model, isotherm—Freundlich model; experiments with real eutrophic lake water—81–100% removal in 24 h (11–69 μg/L initial concentration).	[56]
β-Cyclodextrin	Humic acid, 10 mg/L	Biostimulant	β-cyclodextrin polymer synthesized in the aqueous phase using tetrafluoroterephthalonitrile as a rigid crosslinker, epichlorohydrin as a flexible crosslinker and 2,3- epoxypropyltrimethylammonium chloride as a quaternization reagent	Maximum adsorption 40 mg/g	Solid/liquid ratio = 1 mg/mL; adsorption superior to commercial materials; kinetics—pseudo-second-order model and Elovich model (solute adsorption rate decreases with the increase of adsorbed solute); isotherm—Freundlich model; no significant adsorption decrease after five recycling cycles	[57]
β-Cyclodextrin	Fluvic acid, 30 mg/L	Biostimulant		Maximum adsorption 166 mg/g		
β-Cyclodextrin	2,4,6-trichlorophenol, 0.1 mmol/L	Fungicide, herbicide, insecticide		Maximum adsorption 108 mg/g		
Chitosan, gelatin	Atrazine (20 mg/L), fenitrothion (20 mg/L)	Pesticides	Polymeric composite prepared by inotropic gelation at room temperature	Adsorption capacity 75.19 mg/g (atrazine), 36.23 mg/g (fenitrothion)	Composite (0.3 g/L) added to 50 mL pollutant solution; adsorption time 180 min. Isotherm—Langmuir model	[58]
Cellulose	Chlorpyrifos, 100 μg/L	Insecticide	Polymeric films developed by incorporating dibutyl sebacate, bis(2-ethylhexyl) sebacate, bis(2-ethylhexyl) phthalate, bis(1-butylpentyl) adipate, 2-nitrophenyl octyl ether or 2-fluorophenyl 2-nitrophenyl ether in cellulose triacetate, by solvent casting	Extraction efficiency from synthetic water 71–87% (after 8 h)	Best results obtained for composite with bis(2-ethylhexyl) sebacate; water samples maintained in contact with the film having area of 2.89 cm^2^, under orbital agitation	[59]
β-cyclodextrin	Humic acid	Biostimulant	Friedel–Crafts alkylation reaction between modified β-cyclodextrin and 4,4′ -bis(chloromethyl)-1,1′—biphenyl in a homogeneous ionic liquid system	Maximum adsorption 142 mg/g	0.015 g of composite dispersed in 15 mL of 20 mg/L pollutant; results superior to activated carbon; isotherm—Freundlich model; over 90% efficiency after six recycling cycles	[60]

**Table 4:** Management of industrial dyes and dye models using natural polymers (in chronological order).

Natural Polymer	Targeted Application	Application Form	Obtained Results	Process Parameters	Ref.
Cellulose	Adsorption of crystal violet (dye with practical applications), 11.1 mg/L	Freeze-dried foams consisting of cellulose nanofibers (obtained by addition of epoxypropyl trimethyl ammonium chloride at 65 °C, 8 h) with adsorbed humic acid	55% adsorption	Porous foam with density of 25 kg/m^3^, porosity 98% (20 mg) added to 45 mL of dye solution	[53]
Chitosan	Reduction of methylene blue (model dye), 10 ppm	Silver nitrate mixed with chitosan/polyethylene glycol solution, various concentrations of TiO_2_ added at 80 °C	63.48% degradation	Direct sunlight photocatalysis; Langmuir–Hinshelwood mechanism	[65]
Xylan	Adsorption of methylene blue (model dye), 400 mg/L	Xylan/poly(acrylic acid) magnetite nanoparticles nanocomposite hydrogel	Maximum adsorption capacity—438.60 mg/g	Removal rate >90% for 3 g/L adsorbent; isotherm—Langmuir model; kinetics—pseudo-second-order model	[66]
Cellulose and chitosan	Adsorption of Congo red (histological staining agent), 30 mg/L	Cellulose/chitosan hydrogel prepared by extruding and regenerating from ionic liquid 1-ethyl-3-methylimidazolium acetate in ethanol	Maximum adsorption capacity—40 mg/g	For adsorbent dosage of 2.0 g/L, equilibrium was reached within 115 min, removal rate was 89.6%; isotherms—Langmuir model; kinetics—pseudo-second-order model	[67]
Silk	Filtration of dyes with industrial and biotechnology applications	Membranes prepared by vacuum filtration of exfoliated degummed *Bombyx mori* silk fibers	>90% rejection	Vacuum filtration device, best results obtained for Alcian Blue 8GX (100%, initial concentration 185 µM), Brilliant Blue G (100%, initial concentration 398 µM), Rhodamine B (91%, initial concentration 5 mM)	[68]
Chitosan	Photodegradation of Ponceau BS (staining agent)	Polyaniline-grafted chitosan prepared by chemical using ammonium per sulfate; Ag nanoparticles incorporated into the polymer matrix	Complete degradation after 40 min	Photodegradation under visible light; kinetic—pseudo-first-order model	[69]
Chitosan, lignin	Removal of acid black-172 (dye with industrial applications), 100 mg/L	Ternary graft copolymer (chitosan–acrylamide–lignin), synthesized using microwave irradiation and chemical-free radical initiator technique	Removal efficiency—97.1%	Dosage 200 mg/L; possible mechanisms—charge neutralization, bridging and sweeping effects	[70]
Guar gum	Catalytic oxidation of methyl orange (100 ppm)	Zerovalent iron–guar gum nanocomposite, spherical, particle size of ∼60–70 nm (0.5 g/L)	99% after 60 min, pH 7	Superior oxidation to zerovalent ion by itself (39%)	[41]
β-cyclodextrin	Adsorption of methylene blue (model dye), 100 mg/L	Citric acid-crosslinked β-cyclodextrin	Maximum adsorption capacity—0.9229 mmol/g	0.4 g in 200 mL pollutant, pH = 1–10; kinetics—pseudo-second-order model; isotherm—Sips model; no decrease in performance after five recycling cycles	[71]
Lignin	Adsorption of methylene blue (model dye), 1 mg/mL	Hydrogels obtained by crosslinking poly(methyl vinyl ether co-maleic acid) and lignin in ammonium and sodium hydroxide solutions	Adsorption capacity—629 mg/g	Dry hydrogels (20–30 mg) placed in 20 mL pollutant solution, stirred for 48 h at room temperature; maximum removal efficiency—96%, superior results to control hydrogels (without lignin)	[72]
Pectin	Adsorption of methylene blue (model dye), 100–1000 mg/L	Pectin microgel particles	Adsorption capacity—284.09 mg/g	Different uptake times (2–310 min), pH 1–7; isotherm—Langmuir model; kinetics—pseudo-second-order model; recovery efficiency higher than 80% after three cycles	[73]
Chitosan	Adsorption of CI Basic Red 14 (dye with industrial applications), 100 ppm	Polymeric beads containing chitosan, *Arundo donax* L. cells, gelatin and poly(vinyl)pyrrolidone	Maximum adsorption capacity—41.322 mg/g	Removal efficiency of 92.2% (at 2 g. adsorbent); isotherms—Langmuir model; kinetics—pseudo-first-order model	[74]
(Konjac) glucomannan	Adsorption of malachite green (common dye with industrial applications)	Konjac glucomannan/graphene oxide sponges prepared by ice template method	Maximum adsorption capacity—189.96 mg/g	Isotherms—Langmuir model; kinetics—pseudo-first-order model; adsorption capacity relatively high after five recycling cycles	[75]
Sodium alginate	Adsorption and photocatalytic degradation of crystal violet (dye with practical applications)	Grafted sodium alginate/ZnO/graphene oxide composite	Maximum adsorption capacity—13.85 mg/g	Maximum capacity at pH = 5; isotherm—Freundlich model; kinetics—pseudo-second-order model; photocatalytic degradation enhanced the removal efficiency by 10%	[76]
Tragacanth gum	Adsorption of methylene blue (model dye)	Hydrogel nanocomposite composed of tragacanth gum and modified CaCO_3_ nanoparticles	Maximum adsorption capacity—476 mg/g	Isotherm—Langmuir model; kinetics—pseudo-first-order model; film diffusion—main mechanism of adsorption	[77]
Chitosan	Adsorption of Acid Blue-113 (industrial dye)	Chitosan-coated polyacrylonitrile nanofibrous mat	Maximum adsorption capacity—1708 mg/g	Superior results compared with control (without chitosan); superior capacity than commercial activated carbon; isotherm—Langmuir model; kinetics—pseudo-second-order model; film diffusion—main mechanism of adsorption; slight decrease in adsorption after four cycles	[78]
Silk	Adsorption of crystal violet (dye with practical applications)	3D porous network in a freeze-dried silk fibroin/ soursop seed polymer composite	Maximum adsorption capacity—83.31 mg/g	Isotherms—Freundlich model; kinetics—pseudo-first-order model.	[79]
Sodium alginate,	Adsorption of crystal violet (cationic dye with practical applications), 200 mg/L	Hydrogel beads from rice bran combined with sodium alginate	Maximum adsorption capacity—454.55 mg/g	Isotherms—Freundlich model; kinetics—pseudo-second-order model; no adsorption decrease after five regeneration cycles	[80]
Chitosan	Adsorption of reactive blue 4 (anionic dye with practical applications), 200 mg/L	Hydrogel beads from rice bran combined with chitosan	Maximum adsorption capacity—212.77 mg/g	Isotherms—Langmuir model; kinetics—pseudo-first-order model; satisfactory adsorption (20% decrease) after five recycling cycles	
Sodium alginate	Degradation of methylene blue (model dye)	Ca-alginate/CuO beads	92% degradation in 8 min	100 mg composite added to 5 mL methylene blue solution in the presence of 1 mL NaBH_4_ (0.08 mol/L); kinetics—pseudo-first-order model; degradation reduced when using recycled composites; slow decrease in degradation after ten recycling cycles	[81]
Chitosan	Adsorption of Arsenazo-III (staining, analytical reagent), 100 mg/L	Chitosan hydrogel polymer, initiator potassium persulphate	Maximum adsorption capacity—99.9 mg/g	pH = 6, shaking time of 120 min, polymer dose of 0.01 g, room temperature	[82]
Chitosan	Alizarin Red S (staining, analytical reagent), 100 mg/L		Maximum adsorption capacity—62.5 mg/g		
Sodium alginate	Separation of Congo red (histological staining agent), 0.1 mg/L	Nacre-inspired multiple crosslinked polyvinyl alcohol/ calcium alginate/SiO_2_ membrane	Rejection efficiency—99.5%	Long-term separation properties demonstrated by the membrane; over 99% of initial efficiency retained after three recycling cycles	[83]
	Separation of Alizarin red (staining, analytical reagent), 0.1 mg/L		Rejection efficiency—99.1%		
	Separation of Sunset yellow (dye for food and beverage industry), 0.1 mg/L		Rejection efficiency—98.3%		

**Table 5:** Management of other types of EPs from industrial sources using natural polymers (in chronological order).

Natural Polymer	Targeted Application	Pollutant Class	Application Form	Obtained Results	Process Parameters	Ref.
Chitosan	Adsorption of chlorophenol (50 mg/kg) and phthalic anhydride (70 mg/kg)	industrial precursor/plasticizer	Poly(N,N-diethylacrylamide), poly(N-isopropylacrylamide) and poly(N-vinylcaprolactum) grafted on chitosan/derivatives	100% removal of organic impurities	Best results obtained for poly(N-isopropylacrylamide) graft carboxymethylchitosan at 6 mg/mL; materials can be used for at least 5 cycles	[86]
Cellulose	Flocculation of nonylphenol, 100 μg/L	Precursor for antioxidants, lubricating oil additives, laundry, dish detergents, emulsifiers, solubilizers, surfactants	(poly N-isopropyl acrylamide)-co-(poly diallyl dimethyl ammonium chloride) grafted on carboxymethylcellulose	Flocculation, nonylphenol removal = 79%	Optimized conditions: pH = 4; T = 35 °C; dosage = 40 mg/L	[87]
β-cyclodextrin	Adsorption of perfluorooctanoic acid	surfactant, industrial importance	β-cyclodextrin decafluorobiphenyl polymer (DFB-CDP, 1:β-CD feed ratio = 3)	Adsorption capacity = 34 mg/g, superior to sieved coconut shell-activated carbon	Freundlich model best fit the adsorption isotherm; no significant differences after four recycling cycles	[88]
β-cyclodextrin	Adsorption of bisphenol A, 100 mg/L	Plastics industry	Citric acid-crosslinked β-cyclodextrin	Maximum adsorption capacity = 0.3636 mmol/g	0.4 g in 200 mL pollutant, pH = 1–10; kinetics—pseudo-second-order model; isotherm—Langmuir model; 80% adsorption capacity after five recycling cycles	[71]
β-Cyclodextrin	Adsorption of 2-naphthol, 0.1 mmol/L	Intermediate in dyes production	β-cyclodextrin polymer synthesized in the aqueous phase using tetrafluoroterephthalonitrile as a rigid crosslinker, epichlorohydrin as a flexible crosslinker and 2,3- epoxypropyltrimethylammonium chloride as a quaternization reagent	Maximum adsorption = 74 mg/g	Solid/liquid ratio = 1 mg/mL; adsorption superior to commercial materials; kinetics—pseudo-second-order and Elovich (solute adsorption rate decreases with the increase of adsorbed solute) models; isotherm—Freundlich model	[57]
β-Cyclodextrin	Adsorption of 3-phenylphenol, 0.1 mmol/L	Colorimetric reagent		Maximum adsorption = 101 mg/g		
β-Cyclodextrin	Adsorption of bisphenol A, 0.1 mmol/L	Plastics industry		Maximum adsorption = 103 mg/g		
β-Cyclodextrin	Adsorption of bisphenol S, 0.1 mmol/L	Industrial application in epoxy resins		Maximum adsorption = 117 mg/g		
Chitosan	Catalytic reduction of p-nitrophenol, 5 mM	Industrial intermediate	Chitosan/reduced graphene oxide-based composite hydrogel, with glutaraldehyde crosslinking agent, loaded with Pd nanoparticles	Catalytic reduction, rate constant = 0.0348 min^−1^	Hydrogel mixed with 1 mL pollutant solution, added 10 mL NaBH_4_ solution (0.01 M); results superior to literature data, better for 2-nitroaniline	[89]
Chitosan	Catalytic reduction 2-nitroaniline, 5 mM	Industrial intermediate		Catalytic reduction, rate constant = 0.125 min^−1^		
Chitosan	Degradation of phenol, 100 mg/L	Industrial intermediate	Recombination of chitosan and polyvinyl alcohol, with microorganisms added	Biodegradation, 99%, degradation rate increasing after several uses	Best results obtained at 30 °C, pH = 7, higher compared with microorganism alone; over 95% degradation rates after 90 recycling cycles	[90]
Chitosan	Degradation of p-cresol, 100 mg/L	Industrial intermediate		Degradation in the presence of 1600 mg/L phenol, concentration decreased to 0.4 mg/L		
Chitosan	Degradation of catechol, 100 mg/L	Industrial intermediate		Degradation in the presence of 1600 mg/L phenol, concentration decreased to 0.6 mg/L		
Chitosan	Degradation of 2-aminophenol, 100 mg/L	Industrial intermediate		Degradation in the presence of 1600 mg/L phenol, concentration decreased to 4.7 mg/L		
Chitosan	Degradation of p-nitrophenol, 10 mg/L	Industrial intermediate	Bi-functional ternary nanocomposite constructed using chitosan-wrapped carbon nanofibers, embedded with Ag-doped Co_3_O_4_	Degradation constant = 0.0186 min^−1^; degradation efficiency = 97.39% for 40 mg catalyst	10–40 mg catalyst immersed in 100 mL pollutant solution; solution irradiated with visible light; degradation rate higher than carbon nanofibers and metal oxide alone; over 90% degradation after five recycling cycles	[91]
β-cyclodextrin	Adsorption of bisphenol A, 20 mg/L	Plastics industry	Friedel–Crafts alkylation reaction between modified β-cyclodextrin and 4,4′-bis(chloromethyl)-1,1′-biphenyl in a homogeneous ionic liquid system	Maximum adsorption = 257.75 mg/g	0.015 g of composite dispersed in 15 mL pollutant; results superior to activated carbon; isotherm—Freundlich model; over 90% efficiency after six recycling cycles	[60]
Sodium alginate	Degradation of p-nitrophenol, 10^−4^ M	Industrial intermediate	Ca-alginate/CuO beads	Degradation constant = 0.202 min^−1^; degradation efficiency = 97–98%	100 mg of composite, in the presence of 0.08 mol/L NaBH_4_. Degradation efficiency higher than Ca-alginate alone; kinetics—pseudo-first-order model; 75% reduction after 60 min, after 10 recycling cycles	[81]
Chitosan	Degradation of p-nitrophenol, 20 mg/L	Industrial intermediate	Chitosan/Ag nanoparticles/layered double-hydroxide nanocatalyst	Apparent rate constant = 1.65 × 10^−2^ s^−1^	20 mg nanocatalyst added to 4 mL NaBH_4_ (0.54 g/L) and 6 mL of pollutant solution; kinetics—pseudo-first-order model; performance unchanged by 5 recycling cycles	[92]
Cellulose	Extraction of triclosan, tonalide, 100 μg/L	Personal care products	Polymeric films developed by incorporating dibutyl sebacate, bis(2-ethylhexyl) sebacate, bis(2-ethylhexyl) phthalate, bis(1-butylpentyl) adipate, 2-nitrophenyl octyl ether or 2-fluorophenyl 2-nitrophenyl ether in cellulose triacetate, by solvent casting	Extraction efficiency from synthetic water = 68–93%/44–94% (after 8 h)	Best results for composite with 2-nitrophenyl octyl ether/dibutyl sebacate; water samples maintained in contact with the film having area of 2.89 cm^2^, under orbital agitation	[59]

**Table 6:** Management of other hazardous EPs using natural polymers (in chronological order).

Natural Polymer	Targeted Application	Pollutant Class	Application Form	Obtained Results	Process Parameters	Ref.
Silk	Filtration of gold nanoparticles, 5 nm, 5.5 × 10^13^ unit per mL	Nanoparticles	Membranes prepared by vacuum filtration of exfoliated degummed *Bombyx mori* silk fibers	Rejection 99%	Vacuum filtration device	[68]
	CdSeS/ZnS quantum dots, 6 nm, 1 mg/mL			Rejection 100%		
Cellulose	Removal of pyrene (25, 100 ppb) from water	Polycyclic aromatic hydrocarbon	Cellulose fibers grafted with poly(lauryl acrylate) and poly(octadecyl acrylate	Adsorption capacity 38 mg/g	Higher capacity for octadecyl acrylate grafted cellulose	[99]
(Konjac) glucomannan	Adsorption of U (VI)	Radionuclides	Konjac glucomannan/graphene oxide sponges prepared by ice template method	Maximum adsorption capacity—266.97 mg/g	Selectivity in multi-ions system; isotherms—Langmuir model; kinetics—pseudo-first-order model; adsorption capacity relatively high after five recycling cycles	[75]
Cellulose	Removal of fluorene, 20 µg/L	Polycyclic aromatic hydrocarbon	Polystyrene–cotton composites, obtained by dipping pretreated cotton in 2% polystyrene solution in chloroform	Extraction recovery after two elution = 65%	Recovery increased at the second elution	[100]
	Removal of anthracene, 20 µg/L			Extraction recovery after two elution = 71%		
	Removal of fluoranthene, 20 µg/L			Extraction recovery after two elution = 85%		
	Removal of pyrene, 20 µg/L			Extraction recovery after two elution = 93%		
Cellulose	Removal of polycyclic aromatic hydrocarbons from mainstream cigarette smoke	Polycyclic aromatic hydrocarbon	Cellulose cigarette filter with porous structure, pore size controlled by polyvinylpyrrolidone using a dip–dry method	Removal efficiency 61.79%	Higher than conventional cellulose acetate filter (39.22% removal)	[101]
Cellulose	Plastic micro/nanoparticles stained with neutral red dye	Microplastic particles from commercial body scrub	Cellulose surface functionalized with polyethylenimine	Maximum adsorption efficiency of 97%, 98% and 99% for polymethyl methacrylate, polyvinyl chloride and polyvinyl acetate nanoparticles	Kinetics—pseudo-second-order model	[102]
Cellulose, β-cyclodextrin	Adsorption of microcystin-LR, 1.5, 0.8 µg/mL	Cyanotoxin	Aerogels from cellulose nanofibril grafted with β-cyclodextrin	Adsorption capacity 0.078 mg/g	Aerogels placed in 20 mL of toxin solutions at room temperature and constant stirring; kinetics—pseudo-second-order model	[103]
Sodium alginate	Adsorption of U (VI), 5 mmol/L	Radionuclides	Granulated resin obtained by crosslinking sodium alginate with 1,6-hexamethylene diisocyanate in benzene	Maximum adsorption capacity—269.80 mg/g	Replacement of Na^+^ ions by H^+^ (using dilute mineral acids); replacement of hydrogen ions with metal ions by ion exchange. Higher capacity for U ions compared to other metals	[104]
Polysaccharides	Coagulation and removal of TiO_2_ nanoparticles in the form of TiO_2_–humic acid complex	Nanoparticles	*Enteromorpha* *prolifera* polysaccharide together with poly aluminum chloride	Removal efficiency 87.12%	Highest efficiency for 1 mg/L polysaccharides; in the presence of poly aluminum chloride 95.68%; isotherm—Langmuir model; faster growing, larger and stronger flocs	[105]

## Data Availability

Not applicable.
